# Preservation of Dental Structure in Prosthetic Restorations: Vertical and Horizontal Preparation Techniques. Systematic Review

**DOI:** 10.1002/cre2.70360

**Published:** 2026-04-20

**Authors:** Maria Lisa Florescu, Gelsy Arrien‐Barrenetxea, Luca Fiorillo, Artak Heboyan, Cosimo Galletti, Santi Costa‐Palau, Francisco Real‐Voltas

**Affiliations:** ^1^ School of Dentistry, Department of Integrated Dentistry International University of Catalonia (UIC) Barcelona Spain; ^2^ Department of Medicine and Surgery University of Enna “Kore” Enna Italy; ^3^ Department of Dental Research Cell, Dr. D.Y. Patil Dental College & Hospital Dr. D. Y. Patil Vidyapeeth (Deemed to be University) Pune India; ^4^ Department of Research Analytics, Saveetha Dental College and Hospitals, Saveetha Institute of Medical and Technical Sciences Saveetha University Chennai India; ^5^ Department of Prosthodontics, Faculty of Stomatology Yerevan State Medical University after Mkhitar Heratsi Yerevan Armenia; ^6^ Department of Prosthodontics, School of Dentistry Tehran University of Medical Sciences Tehran Iran; ^7^ Department of Prosthodontics International University of Catalonia (UIC) Barcelona Spain; ^8^ Department of Integrated Dentistry International University of Catalonia (UIC) Barcelona Spain

**Keywords:** BOPT, BOPTm, conservative, horizontal preparations, tooth preparation, tooth structure removed, vertical preparations

## Abstract

**Objectives:**

Crown preparation leads to irreversible dental tissue loss, which affects long‐term restoration success. The advantages of horizontal (chamfer) versus vertical preparation techniques (BOPT, BOPTm) remain debated. This systematic review evaluates which method is most conservative regarding dental structure removal and examines their clinical indications, advantages, and limitations.

**Materials and Methods:**

This systematic review followed the Cochrane Handbook and PRISMA 2020 guidance. The electronic search was performed in PubMed, Cochrane and Web of Science for studies published between 2012 and 2023. The PROSPERO record (CRD420251161178; registered on 06 October 2025) was retrospective. Clinical studies, in vitro/ex vivo investigations and contextual narrative sources were included for different purposes. The >=6‐month follow‐up criterion was applied only to longitudinal clinical outcome studies; laboratory and descriptive studies were included only for mechanistic or contextual evidence. The primary outcome was direct or indirect quantification of tooth‐structure removal. Secondary outcomes included periodontal health, function, tooth morphology, esthetics, and pulpal implications. Certainty of evidence was evaluated using the GRADE methodology.

**Results:**

Twenty‐three studies were included. Six studies directly quantified tooth‐structure removal or residual dentin thickness using gravimetric analysis, micro‐CT, digital volumetric analysis, or related methods. Across studies that compared preparation geometries at a comparable margin level, BOPT was generally more conservative than BOPTm; however, supragingival or juxtagingival horizontal chamfer preparations remained the most conservative option when margins could be maintained outside or at the gingival sulcus. Periodontal advantages of BOPT were mainly reported for subgingival clinical scenarios.

**Conclusions:**

The apparent difference between vertical and horizontal preparations is scenario‐dependent. When a subgingival restorative margin is required, BOPT may preserve more tooth structure than BOPTm and may offer favorable periodontal behavior compared with subgingival horizontal preparations. When margins can be placed supra‐ or juxtagingivally, horizontal chamfer preparations remain the most conservative and clinically predictable approach.

## 1 Introduction

Conservative dentistry refers to a treatment philosophy prioritizing the preservation of natural teeth and healthy dental structure to the greatest extent possible. Full coverage fixed prosthodontic restorations aim to restore lost health, function, and esthetics while protecting the tooth and surrounding soft tissues. These restorations must harmonize with adjacent teeth, and their margins should remain stable over time (Molina et al. 2019). The success of a prosthetic crown is defined by marginal adaptation, esthetics, and fracture resistance. The loss of extensive dental structure may weaken the tooth and increase susceptibility to fractures (Łabno and Drobnik 2020). Preserving dental structure redistributes occlusal forces, thus reducing the risk of fracture. Therefore, it can be stated that the less dental structure that is removed, the greater the likelihood of success of a prosthetic crown.

A major debate in current prosthodontics revolves around horizontal versus vertical tooth preparation, with or without finish line. This debate encompasses various factors, including marginal fit and sealing, vertical seating, gingival margin location in horizontal preparations, periodontal health, apical migration of periodontal tissues over time, among others. It also includes which technique is more conservative regarding dental structure removal. According to literature, vertical preparation preserves more dental structure than horizontal preparations (Łabno and Drobnik 2020; Loi and Di Felice 2013; Kaur et al. 2022).

The vertical or marginless preparation technique was introduced in 1974 at the University of Pennsylvania, primarily targeting patients with periodontitis. It has gained prominence in recent years, particularly with Ignacio Loi's Biologically Oriented Preparation Technique (BOPT) protocol (Molina et al. 2019). Currently, vertical preparation techniques include BOPT and its modified version (BOPTm). BOPT aims to eliminate the volumetric emergence component of tooth anatomy through vertical knife‐edge preparation, allowing the restoration margin to be set by the laboratory rather than the dentist (Loi and Di Felice 2013) in order to achieve prosthetic dominance. The BOPT technique is not only a dental preparation method but also offers biological advantages such as increasing periodontal tissue thickness, providing stability to the gingival margin, and achieving gingival remodeling by altering the emergence situations of the provisional prosthetic crown (Kaur et al. 2022). This is a dynamic process and an interaction between the prosthesis and periodontal tissues.

The modified BOPT technique (BOPTm), introduced by Vela and Rodriguez (Rodríguez et al. 2019), follows the biological concepts of BOPT by utilizing the bone crest as the limit for vertical tooth preparation. With this modification, the periodontal regeneration obtained at the end is thicker, and it's also possible to establish the periodontal tissues at a more coronal level compared to where they were previously (Rodríguez et al. 2019). This technique may be considered more aggressive in conservative terms compared to BOPT. In addition, it requires precise execution to ensure predictable outcomes.

Horizontal preparation techniques or with a finish line involve creating a termination line on the tooth where the prosthesis can be seated. Unlike vertical preparations, in this case, the termination line is created by the operator, and there may be more technical difficulties, as it depends on the operator's experience. The location of the margins can be supragingival, juxta gingival, or subgingival. Many authors agree that supragingival margin placement is most compatible with periodontal health (Molina et al. 2019) and likely the most conservative method. However, due to old restorations, subgingival caries, or esthetic needs, it may be necessary to place the margins within the gingival sulcus (Molina et al. 2019). Horizontal preparations have demonstrated predictability and durability over time; however, problems with apical migration of the periodontal tissues have been described when placing margins subgingivally, as the gingiva follows the tooth. These successive apical migrations of periodontal tissues can cause serious esthetic issues, sensitivity problems, and caries (Loi and Di Felice 2013), ultimately leading to treatment failure.

The main objective of this article is to determine which dental preparation technique is more conservative, in terms of eliminated dental structure, depending on whether working with BOPT, BOPTm, and chamfer, through a systematic literature review. The secondary objectives of this article are to assess the advantages and disadvantages of the mentioned preparation techniques in terms of periodontal health, function, esthetics, and tooth morphology.

## 2 Materials and Methods

The report of this systematic review has been guided by the Cochrane checklist (Higgins et al. 2023) and the PRISMA 2020 guidelines (Page et al. 2021). The review protocol was registered in the PROSPERO database (CRD420251161178) on 06 October 2025. This registration was retrospective relative to the conduct of the review. The review was originally developed as an academic Final Degree Project. During preparation of the manuscript for journal submission, the authors retrospectively registered the review to improve transparency and explicitly acknowledge this timing. The review question, eligibility criteria, search sources, primary and secondary outcomes, and qualitative synthesis framework had been prespecified in the academic working protocol used for the thesis before screening; however, the public PROSPERO registration was finalized after screening and data extraction. Accordingly, the review should not be interpreted as prospectively registered. No quantitative meta‐analysis had been planned due to expected heterogeneity in study designs, outcomes, and measurement methods. The review question was structured according to PICOS: in patients requiring a fixed dental prosthesis as restorative treatment (P), does the Biologically Oriented Preparation Technique (BOPT) (I) provide superior conservation of dental structure compared with modified BOPT or conventional horizontal/chamfer finish lines (C), when assessed through direct quantitative tooth‐reduction measures and clinical periodontal/restorative outcomes (O), across available clinical, laboratory, and contextual evidence (S)?

A comprehensive search was carried out in PubMed, Cochrane, and Web of Science between September 2023 and February 2024 for studies published between 2012 and 2023. Search strategies combined free‐text terms and, when applicable, MeSH terms related to tooth preparation, margin design, periodontal tissues, and restoration. The complete search strings are reported in Tables 1 and 2. The search, screening, data extraction, and appraisal were conducted by one reviewer because this review was developed within the time constraints of the Final Degree Project; this is acknowledged as a methodological limitation.

**Table 1:** Research strategy for systematic review.

Electronic databases and libraries	Search terms, MeSH terms, and Boolean operators
PubMed	− (“Dental Margins, Restoration”[MeSH] OR “Subgingival” OR “Periodontal Diseases”[MeSH]) − (“Tooth Preparation”[MeSH]) AND (“Vertical” OR “Horizontal”)
Cochrane	− (Horizontal dental preparations) AND (Vertical dental preparations) AND (Randomized controlled trial) − (Volumetric analysis) AND (Tooth structure removed)
Web of science	− BOPT, BOPTm − (“Tooth Preparation”[MeSH]) AND (finish lines) AND (conservative) (Finish lines) AND (Conservative) − (Tooth preparation) AND (Tooth structure removed)

**Table 2:** Results of the search and articles included in the systematic review.

Search terms and Boolean operators	No. of found articles	No. of chosen articles
(“Dental Margins, Restoration”[MeSH] OR “Subgingival” OR “Periodontal Diseases”[MeSH])	168	3
(“Tooth Preparation”[MeSH]) AND (“Vertical” OR “Horizontal”)	82	2
(Horizontal dental preparations) AND (vertical dental preparations) AND (randomized controlled trial)	4	1
(Volumetric analysis) AND (Tooth structure removed)	16	3
BOPT, BOPTm	40	3
(“Tooth Preparation”[MeSH]) AND (finish lines) AND (conservative)	9	4
(finish lines) AND (conservative) AND (randomized controlled trial)	1	1
(tooth preparation) AND (tooth structure removed)	347	6

The primary outcome was preservation of dental structure, defined as direct quantitative measurement of removed hard tissue (e.g., percentage weight loss, volumetric reduction, or residual dentin thickness) or, when direct quantification was unavailable, the closest reported proxy. Secondary outcomes included periodontal health (probing depth, plaque/inflammation, gingival margin stability, recession, CAL when available), functional performance, esthetics, tooth morphology, and pulpal implications.

The intervention under study was BOPT, including modified BOPT (BOPTm). Comparators included horizontal or chamfer finish‐line designs and other conventional preparation approaches. Because the available literature was sparse and heterogeneous, the review included human clinical studies, animal studies, in vitro/ex vivo studies, systematic reviews, narrative reviews, case reports, and expert opinions; however, these designs were not weighted equally. The requirement for >=6 months follow‐up was applied only to longitudinal clinical studies evaluating clinical outcomes. Laboratory studies, case reports, and contextual narrative sources were included to inform mechanistic or descriptive aspects and were not treated as equivalent evidence for longitudinal clinical effectiveness.

The exclusion criteria were as follows: studies not aligned with the review objectives; studies lacking sufficient methodological detail for extraction; studies that did not include the relevant search concepts; and studies published before 2012, except for seminal methodological papers retained for contextual importance. RoB 2 was used for randomized clinical studies, and ROBINS‐I was used for non‐randomized clinical intervention studies. Narrative reviews, expert opinions, and laboratory studies were discussed descriptively; formal GRADE certainty ratings were restricted to outcome‐level clinical questions and were not used to elevate mechanistic or opinion‐based evidence.

## 3 Results

**Figure 1:**
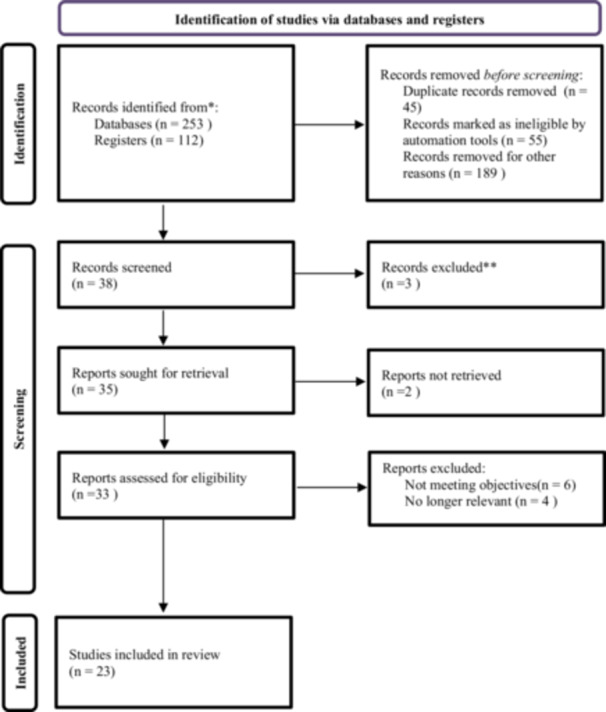
Flowchart: Study selection process following Prisma 2020 (Page et al. 2021) flow diagram.

The electronic database search was conducted in four stages (Figure 1). The use of MeSH terms and Boolean operators allowed for a refined and specific search strategy, yielding a total of 327 records (databases = 253; registers = 74). After removal of 45 duplicates, 55 records were excluded by automation tools, and 189 records were removed for other prespecified reasons, 38 records underwent title/abstract screening. Three records were excluded at this stage. Thirty‐five full‐text reports were sought, 2 were not retrieved, and 33 full‐text reports were assessed for eligibility. Ten reports were excluded at full text: six did not address preservation of tooth structure or relevant periodontal/restorative outcomes, three lacked sufficient methodological detail for data extraction, and one was outside the predefined publication period. Twenty‐three studies were included in the qualitative synthesis. This textual PRISMA accounting is provided to clarify the full‐text exclusions and to resolve inconsistencies in the original figure.

**Figure 2:**
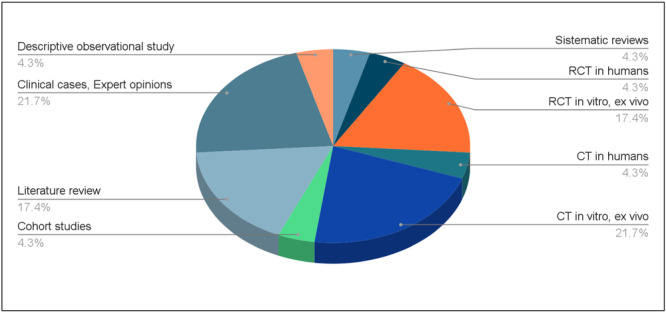
Ordered Pie chart of levels of evidence from most to least, with the respective percentage representing the total of 23 studies used in the review.

**Figure 3:**
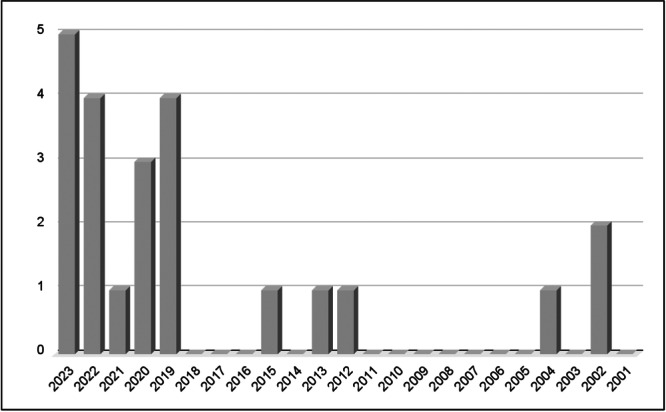
Bar graph showing the publication years of studies included in the review.

The included studies comprised a range of designs, reflecting the multifactorial nature of prosthetic tooth preparation. Specifically, one systematic review (4.3%), five randomized controlled trials (21.7%), six controlled clinical trials (26%), one cohort study (4.3%), and ten descriptive or narrative reports (43.7%), including case reports and expert opinions, were included (Figure 2). Most studies were published between 2018 and 2024, with three seminal studies (2002, 2004) retained for their foundational contribution to the understanding of vertical preparation techniques. Table 3 summarizes the main characteristics of the included studies, including design, publication year, sample, and main findings. The assessment of certainty of the studies has been conducted using the GRADE methodology (Schünemann et al. 2023) which analyzes risk of bias, inconsistency, indirectness, impression and publication bias (Table 3). Quantitative outcomes were primarily reported through volumetric analyses, while qualitative assessments were based on clinical evaluation and expert consensus. Only six studies directly and quantitatively assessed tooth‐structure removal or residual dentin thickness. These six studies used gravimetric analysis, micro‐CT, digital volumetric analysis, or residual dentin mapping and form the core evidence base for the primary outcome. Table 4 provides a summary of the six studies that directly quantified the primary outcome. Importantly, none of these six studies directly compared BOPT, BOPTm, and horizontal chamfer under a uniform experimental protocol. Therefore, the review conclusions on relative conservatism remain based on triangulation of direct quantitative evidence, mechanistic reasoning, and clinical contextual studies rather than on a single head‐to‐head quantitative dataset (Figure 3).

**Table 3:** Summary of studies used in the systematic review and assessment of certainty with GRADE methodology.

Author	Year	Type of study	Objective	Methods	Results	Conclusion	Assessment of certainty GRADE
(1) Molina A	2019	Literature review and case studies	It is necessary to be aware of the therapeutic options provided by periodontics, as most cases of complex rehabilitation often require a multidisciplinary approach	Exploring the relationship between Periodontology and Restorative Dentistry on teeth through the presentation of clinical cases supported by the literature	Knowledge of periodontal anatomy and the impact of occlusal forces on the periodontium are essential for quality and predictable long‐term restorative dentistry. Periodontal plastic surgery techniques, such as crown lengthening and root coverage procedures, are necessary to improve the health of teeth that will be restored with prosthetics.	The provisional phase is crucial for shaping soft tissues and facilitating their healing after surgical procedures and tooth extractions, allowing for satisfactory esthetic outcomes.	Very Low
(2) Łabno P	2020	Literature review	Compare the advantages and disadvantages of various types of dental preparations for different types of crowns, including the modern concept of vertical preparation (verti prep)	The PubMed and Google Scholar databases have been used in the search for review articles; the summarized results are presented.	The choice of the finish line depends on various factors, such as pulp vitality, tooth location, inclination, the material type for the restoration, crown convexity, patient age, and the size of the construction. At the same time, it should be emphasized that in some clinical situations, such as stump discoloration or insufficient visible dental structure after the removal of an old crown, the decision on the type of preparation and/or reconstruction to be performed is made intraoperatively.	There is no universally recommended method for dental preparation for prosthetic crowns, as the choice depends on various clinical factors.	Low
(3) Loi I.	2013	Clinical case	Introducing and demonstrating the successful application of the Biologically Oriented Preparation Technique (BOPT) in the restoration of periodontally healthy teeth.	The application of the BOPT technique in this clinical case has yielded highly positive results. The stability of the soft tissue at the prosthesis/tissue interface has been consistently maintained both in the short and long term. (15‐year follow‐up of the case)	The clinical and esthetic results are remarkable, highlighting the simplicity and effectiveness of the technique compared to other preparation options such as chamfer or shoulder. The BOPT technique proves to be an effective and reliable strategy for the preparation of periodontally healthy teeth.	In the context of prosthetic restoration, the BOPT technique emerges as an attractive and durable option	Moderate
(4) Kaur H	2022	Descriptive study	Explore the technique of vertical preparation without a finish line, in which the existing cement‐enamel junction is removed, and a new junction controlled by the prosthesis margin is established.	The BOPT technique is analyzed and described step by step, both the clinical and laboratory procedures. The advantages, disadvantages, and indications of this technique are also discussed	The vertical preparation without a finish line increases the thickness of the soft tissue, achieving acceptable esthetic results. Clinical trials are needed to validate these practices.”	A high‐quality clinical and esthetic outcome can be achieved regarding the stability of soft tissue at the prosthesis/tissue junction with a minimally invasive approach, preserving biological structures as much as possible.	Low
(5)Rodríguez X	2019	Clinical case	Explore the technique (BOPT) involving vertical preparation and immediate provisionalization, with a focus on understanding the underlying biological mechanism and its impact on periodontal tissue stability.	Histological study in a patient treated with BOPT on teeth 12 and 21. Accidental abutment fracture after 19 months. Knife‐edge carving down to the alveolar crest, dentin exposure. After 19 months, accidental fracture led to considering implant‐supported rehabilitation. Teeth extraction with cone beam computed tomography and prior probing. Preparation of teeth for histological analysis using an optical microscope. Observation of sections stained with hematoxylin‐eosin to evaluate tissue changes.	The human histological study of teeth restored with the BOPT technique revealed regeneration of the supracrestal periodontal ligament in the prepared tooth area, suggesting a possible biological mechanism supporting the stability of periodontal tissues.	Through the application of the BOPT technique and histological observation, a biological reasoning based on the principles of periodontal regeneration is proposed as justification for the stability of periodontal tissues obtained with this technique. New BOPTm technique.	Moderate
(8) Panadero RA	2023	Literature review	Assess the scientific evidence of BOPT on teeth. Its advantages and disadvantages, and the origin and biological basis of the perioprosthetic technique.	Review of published clinical studies on BOPT and subsequent evaluation of its principles, advantages, and disadvantages.	Unlike conventional horizontal preparations, BOPT is expected to provide stability and health to the gingival tissue over time. This restorative philosophy involves a vertical carving without a finishing line associated with rotary curettage of the gingival sulcus stabilized by a prosthetic restoration that simulates the anatomical crown of the natural tooth. There are no clinical studies of more than 6 years. Therefore, long‐term stability is not known.	Scientific evidence has shown that the biologically oriented preparation technique is suitable for use in fixed prosthodontics, demonstrating stability of periodontal tissues and health of the dento‐gingival complex. Long‐term studies are lacking.	Low
(9) Kasem AT	2022	RCT in vitro	Evaluate the fracture resistance of two ceramic systems fabricated using two dental preparation designs (horizontal and vertical prep) using CAD/CAM standardization technology.	Forty human maxillary premolars divided into two groups (H and V) according to preparation technique. Subgroups: CD (Celtra Duo) and K (KATANA) according to material used. Use of CAD/CAM for standardization of dental preparation. 5000 thermal cycles and load until fracture. Evaluation of failure types using stereomicroscopy and scanning electron microscopy (SEM).	The vertical preparation proved to be a promising alternative to horizontal preparation. Additionally, both Celtra Duo and KATANA crowns can be used in the premolar area with a margin thickness of 0.5 mm.	Zirconia‐reinforced ceramic crowns and monolithic zirconia crowns may not require invasive finishing line preparations, as the type of finishing line did not affect the strength after aging conditions.	High
(10) Mancuso E	2020	RCT in vitro	Quantitatively assess the removal of enamel and dentin tissue, as well as the residual adhesive surface (RAS), after using different designs and margin locations for indirect partial restorations (IPR).	A human molar was scanned using Micro‐CT, and the obtained STL file was used to 3D print 50 replicas of dental resin. Standardized preparations were made using IPR, and the samples were randomly assigned to 5 groups according to the preparation and margin location relative to the dental equator (DE). The data on enamel and dentin volume removal were evaluated and statistically analyzed.	When the preparation margin is placed above the DE (dental equator), the beveled joint dental preparation results in greater dentin exposure, reducing the adhesive surface on the enamel. Below the DE, rounded shoulder appears to be more aggressive in terms of tissue removal compared to chamfer.	In teeth requiring partial restoration with the margin below the dental equator, a chamfer preparation would be more conservative than a shoulder preparation. When it is above the equator, flat designs will expose more dentin, providing a less favorable substrate for adhesion.	High
(11) Panadero RA	2019	Expert opinion	Debate between the two specialists Ruben Augustin Panadero and Ernest Mallat on BOPT (vertical) and horizontal dental preparations. A comparison of both techniques is proposed, in which cases one or the other is more recommended, their long‐term predictability, and difficulty.	11 questions are asked to both specialists, covering long‐term success factors of each preparation, comparison of difficulty between techniques, impact of biological space invasion, advantages and disadvantages of each technique… etc. Consequently, each specialist argues their answers to each question with support from clinical cases.	The vertical preparation technique does not replace the horizontal preparation technique, but complements it. The horizontal preparation technique is considered simpler in all aspects, offering excellent results if executed correctly. On the other hand, the vertical preparation technique is especially recommended in fixed prosthesis retreatments with conventional preparations that present adjacent periodontal pathology and in cases of asymmetry in the gingival contour along with old restorations	The future of both techniques will depend on the development of new restoration materials and advancements in digital intraoral scanning processes and digital workflow in prosthetics. The importance of considering the specific indication for each technique is emphasized, with vertical preparation being particularly valuable in retreatments and situations with asymmetries or limited gingiva in abutment teeth.	Very Low
(12) Edelhof D	2002	CT in vitro	Quantify and compare the amount of dental structure removed when various innovative and conventional dental preparation designs were completed on different teeth.	Preparations were made on resin teeth representing the upper left central incisor, upper left canine, and lower left central incisor with different designs: porcelain veneers, resin‐bonded retainers with grooves, all‐ceramic crown, and metal‐ceramic crown. After the dental preparations (10 per group), the crown was separated from the root at the cementoenamel junction (CEJ). The coronal dental structure removed was measured using gravimetric analysis. Means and standard deviations were calculated for the removal of dental structure with different preparation designs	The ceramic veneers and resin‐bonded prosthetic retainers were the least invasive preparation designs, removing approximately 3% to 30% of the coronal dental structure by weight. Approximately 63% to 72% of the coronal dental structure was removed when preparing teeth for all‐ceramic and metal‐ceramic crowns.	The dental preparations for porcelain laminate veneers and resin‐bonded prostheses required approximately between one‐fourth and half of the amount of dental reduction compared to conventional full‐coverage crowns.	Moderate
(13) Davis GR	2012	CT ex vivo	The research has suggested that 2 mm or more of remaining dentin is critical to protect the pulp after dental preparation. The aim of this project was to develop a method to measure the local thickness of dentin after dental preparation for metal‐ceramic crowns.	Microtomography scans (XMT or micro‐CT) of extracted teeth were performed before and after crown preparation. Three‐dimensional maps of dentin thickness coded by colors were generated, and distributions of dentin thickness throughout the tooth were analyzed. This was tested with a single operator on 16 extracted upper central incisors.	Most prepared teeth showed regions with residual dentin thickness less than 1.5 mm; in six cases, it was less than 1.0 mm, and in three of them, it was less than 0.5 mm. This method provides valuable information for assessing preparation technique and instrumentation quality.	Although it is an ex vivo method, it can be used as a research tool to identify patterns of over‐ or under‐preparation, which could lead to potential modifications in technique, instrumentation, and/or crown design.	Moderate
(14) Yu H	2019	Expert opinion	Assess the feasibility and benefits of minimally invasive microscopic dental preparation, exploring its application in dental preservation, periodontal health, and long‐term oral function.	This article aims to illustrate the concept, fundamental elements, and indications of minimally invasive microscopic dental preparation. A clinical protocol for minimally invasive microscopic preparation based on a guideplate of the target restorative space is presented, providing new perspectives on the quantity and shape of minimally invasive microscopic dental preparation	The question is raised as to whether microscopic dentistry offers a significant advantage in dental preservation, pulp protection, and periodontal health.	Although microscopic dentistry shows potential, further research is required to determine its true impact on dental preparation and its clinical benefits	Low
(15) Al‐Fouzan AF	2015	RCT ex vivo	Analyze gravimetrically the dental reduction associated with different commonly used preparation designs in relation to the coronal and radicular parts of the tooth.	Eighty extracted permanent human teeth were divided into eight groups according to tooth type and preparation design. Gravimetric analysis was performed before and after preparation to measure the mass of the removed tooth structure. Descriptive statistics and Student's t‐test were used to compare the dental reduction in percentage of weight, with a significance level of *p* < 0.05.	The full‐coverage preparations, especially for the mandibular first premolars, required the greatest dental reduction (40.01%). In contrast, the lowest dental reduction was observed in the upper central incisors subjected to ceramic veneer preparation (20.19%).	Dental preparations for all‐ceramic crowns require greater dental reduction compared to ceramic veneers and inlays.	High
(16) Sorensen JA	2002	CT in vitro	To quantify the amount of dental structure removed in various innovative and conventional preparation designs for fixed prostheses. Evaluate the impact of different preparation designs on the amount of dental structure removed.	Four Typodont resin teeth representing upper and lower premolars and molars were prepared with various abutment designs (onlay, partial crown, full crown…). (10 per group) The removal of dental structure was measured using gravimetric analysis on a high‐precision scale.	The adhesive and inlay preparation designs turned out to be the least invasive, with removal of dental structure ranging between approximately 5.5% (A2) and 27.2% (I3). Preparations for full crowns proved to be the most invasive, with removal of dental structure varying between 67.5% (F1) and 75.6% (F3).	The adhesive and inlay preparation designs were the least invasive compared to full crown designs. The amount of dental structure removed was also affected by the tooth morphology.	Moderate
(17) Weber AR	2021	CT in vitro	Assess the effect of dental morphology on the amount of dental structure removal and the effect of different evaluation methods on the detected amount of removed dental structure.	Eight test groups (n = 10 each) of standardized artificial teeth for partial and full crowns were prepared. All teeth were prepared by the same operator following predefined preparation parameters. Dental structure removal was measured using three different evaluation methods: digital volumetric analysis (DVA), weight analysis (WA), and computer‐assisted weight analysis (CAWA).	In full crown preparations, both dental morphology (*p* = 0.047) and different evaluation methods (*p* = 0.01) impacted the detected dental structure removal; however, only some comparisons between groups reached the significance level.	The amount of dental structure removal depends on dental morphology and the type of evaluation method, which should be taken into account when comparing results between studies.	Moderate
(18) Mohammad A	2023	RCT in humans	To investigate and compare the influence of subgingival horizontal preparation technique (SHPT) and biologically oriented preparation technique (BOPT) on periodontal health in a split‐mouth model	The sample of 100 patients was divided into two groups using a split‐mouth design; each patient received two crowns, one with the SHPT technique and the other with the BOPT technique. The teeth were randomly assigned. All prepared teeth were restored with zirconia cores and ceramic stratification. Follow‐up checks were performed on the patients at one, three, six months, one year, and two years.	The initial SHPT technique showed better oral health indices, but over time, inflammation and plaque increased, and patient satisfaction decreased. Meanwhile, the BOPT technique had higher probing depth levels at the beginning and at 3 months, but improved over time, showing significant reductions at 6 months, 1 year, and 2 years of follow‐up.	BOPT is a favorable technique with a full crown or a liner, showing good marginal stability and periodontal behavior.	High
(19) Gavelis JR	2004	CT in vitro	Correlate margin design with the placement and sealing of cemented full‐cast crowns under standardized simulated clinical conditions.	Eight stainless steel abutments with coronal preparations similar in size to an average molar. Different margin designs, including scalloped edges, 90‐degree shoulders, and chamfers. Five crowns were fabricated for each abutment and cemented using an Instron testing machine. The cementation pressure was gradually reduced over a 10‐min interval. After cementation, the crowns were embedded in plastic, sectioned, and polished to measure cement spaces.	The feather‐edge (vertical) and chamfer preparations with parallel bevel stood out as the most effective in terms of marginal sealing, while the full 90‐degree shoulder showed the best seating of the restoration.	These findings highlight the importance of considering the marginal design when planning and carrying out full crown restorations.	Moderate
(20) Montañes E	2023	Case study and expert opinion	Report on the paradigm shift in vertical and horizontal preparations. Comparison of techniques.	Presentation of two cases to support statements that gingival recessions should not always be expected when performing preparations with horizontal finishing lines.	The BOPT technique stands out for reducing the likelihood of recessions, showing positive esthetic and gingival outcomes. Although it is true that the risk of recession is higher with horizontal finish lines, the real cause of those recessions needs to be determined.	The key lies in the precise fitting of the crowns, regardless of the orientation of the finish lines, to achieve long‐term stability and avoid gingival problems.	Low
(21) Painz G	2023	Literature review	In selecting the margin design, it's important to identify the appropriate indications and the tooth being treated; patient and clinician characteristics influence this selection.	Review of articles by expert periodontal and prosthetic area for evaluating factors that interfere with the choice of margin design in a full coverage crown. (horizontal vs. vertical). Not considering the BOPT technique.	Due to its technical sensitivity, subgingival vertical preparation must be performed with special care during tooth preparation, provisionalization, communication with the laboratory, cementation, and maintenance. Ideally, for full‐coverage restorations, margins should be placed in a supragingival position to preserve dental structure and not damage periodontal tissues.	In general, in the reality of clinical practice, clinician preferences likely represent the most influential factor when selecting a margin type, as many clinical and technical aspects can significantly affect treatment outcomes.	Moderate
(22) Palombo D	2022	RCT in vivo in animals	Evaluate the healing of hard and soft tissues around teeth prepared with the biologically oriented preparation technique (BOPT) compared to the chamfer technique and unprepared teeth.	Thirty‐two teeth of Beagle dogs were randomly prepared, with 16 using the BOPT technique (test group) and 16 with chamfer (control group). Additionally, 16 negative controls (unprepared teeth) were employed for comparison. Histological descriptions and histomorphometric measurements of periodontal tissues were conducted at 4 and 12 weeks in 7 out of 8 dogs, covering soft tissue height and thickness, as well as horizontal and vertical bone dimensions.	The dental preparation protocols with BOPT and chamfer induced similar qualitative and quantitative changes in the healing of the supracrestal soft tissue complex compared to unprepared teeth.	Despite the limited amount of data, it appears that the differences between the tested preparation techniques were not statistically significant	High
(23) Amesti‐Garaizabal A	2020	CT in vivo in humans	Digitally evaluate the effect of cervical emergence of restorations in terms of gingival tissue volume	Thirty‐one frontal upper teeth (from canine to canine) were used for the study. The BOPT technique was employed, along with different emergence profile angles (30° and 60°) in the provisional process. Each sample was digitized with an intraoral scanner and processed into STL files. A volumetric analysis protocol was conducted using STL file manipulation software. Changes in gingival thickness and position were measured in buccal and lingual positions.	Increasing the emergence profile angle to 60° resulted in a significant increase in gingival thickness on the buccal aspect and in an apical displacement both buccally and lingually. This change was associated with a significant decrease in gingival height.	The soft tissue around the tooth restored with BOPT evolved according to the prosthetic emergence angle of the provisional restoration, facilitating the planning of gingival tissue displacement before the definitive restoration	Moderate
(24) Serra‐Pastor B	2023	Cohort study. Clinical prospective study of 6 years	Assessing the clinical and biological outcomes of fixed partial dentures on teeth prepared using the BOPT technique over a 6‐year follow‐up period.	Zirconia fixed bridges supported by teeth in the anterior region, prepared using the BOPT technique, were evaluated. Participants were monitored annually for 6 years, assessing plaque index, probing depth, vestibular gingival thickness, and gingival margin stability. Biological and/or mechanical complications were recorded, and patient satisfaction was measured using a visual analog scale (VAS)	Twenty‐five fixed partial prostheses in 70 teeth of 24 participants were evaluated. Teeth treated with the BOPT technique showed improved gingival thickness, low levels of plaque, and stable probing depths. The gingival margin remained stable in 100% of cases, and all prostheses had 100% survival rate	Fixed partial dentures supported by teeth prepared using the BOPT technique exhibited good periodontal health, stability in the gingival margin, no recessions, and a 100% survival rate during a 6‐year follow‐up.	Moderate
(25) Abad‐Coronel C	2022	Systematic review	Analyze whether the use of the BOPT technique leads to better clinical outcomes in terms of probing depth, gingival inflammation index, gingival marginal stability, and a lower incidence of mechanical and biological complications.	The PRISMA guidelines were used for the review. An electronic search was conducted in the MEDLINE/PubMed, EMBASE, Sciencedirect, Wiley Online Library, Cochrane, and ProQuest databases for articles published between March 2010 and July 2021 using keywords. Three reviewers selected and analyzed all articles mentioning the BOPT technique and meeting the inclusion criteria.	According to these studies, among all teeth treated with the biologically oriented preparation technique, the probing depth (greater than 3 mm) increased by only 2.3%, gingival inflammation was present in 22.8%, gingival recession occurred in 1.7% (resulting in decreased gingival stability), and mechanical and biological failures were recorded in 4.4% of the teeth	The biologically oriented preparation technique for fixed dental prostheses showed satisfactory clinical outcomes up to 5 years, with low rates of probing depth, gingival inflammation, recession, and mechanical and biological failures.	High

**Table 4:** Studies directly quantifying tooth‐structure removal and residual dentin outcomes.

Study	Measurement method	Sample	Main quantitative finding	Uncertainty/notes
Mancuso et al. (2022)	Micro‐CT/digital volumetric analysis	50 standardized resin replicas	Below the dental equator, chamfer was more conservative than rounded shoulder; above the equator, flatter designs exposed more dentin and reduced residual enamel adhesive surface.	Exact pooled effect sizes were not reported in the extracted manuscript tables.
Edelhoff and Sorensen (2002)	Gravimetric analysis	Anterior typodont resin teeth, 10 per group	Approximately 63% to 72% of coronal tooth structure was removed for all‐ceramic and metal‐ceramic crowns; veneers and resin‐bonded preparations removed about 3% to 30%.	Means and SDs were reported in the source study, but detailed values were not extracted into the manuscript.
Davis et al. (2012)	Micro‐CT residual dentin thickness mapping	16 extracted maxillary central incisors	Most prepared teeth had regions with residual dentin thickness < 1.5 mm; 6 had areas < 1.0 mm and 3 had areas < 0.5 mm.	This was an indirect quantitative proxy of over‐reduction rather than direct volumetric loss.
Al‐Fouzan (2016)	Gravimetric analysis	80 extracted permanent teeth	Mandibular first premolars showed the greatest reduction (40.01%); ceramic veneer preparation of maxillary central incisors showed the lowest reduction (20.19%).	Comparative percentages were available; CI/SD values were not extracted into the review table.
Edelhoff and Sorensen (2002)	Gravimetric analysis	Posterior typodont resin teeth, 10 per group	Adhesive and inlay designs removed about 5.5% to 27.2%; full‐crown designs removed about 67.5% to 75.6%.	Directly quantitative, but not specific to BOPT/BOPTm.
Weber et al. (2022)	Digital volumetric analysis, weight analysis, and CAWA	80 standardized artificial teeth	For full crowns, both tooth morphology (*p* = 0.047) and the evaluation method (*p* = 0.01) significantly influenced the detected amount of tooth reduction.	Effect magnitude depended on the measurement technique; no single pooled estimate was possible.

Periodontal outcomes were reported mainly by one split‐mouth randomized clinical trial, one prospective cohort study, one animal histologic trial, and one prior systematic review of BOPT outcomes (Table 5). Restorative materials, CAD/CAM workflows, and scanning/impression protocols were reported inconsistently and could not be meaningfully stratified across the dataset; this is now stated explicitly as a limitation of the synthesis. Because of design heterogeneity and non‐uniform outcome reporting, a meta‐analysis was not feasible.

**Table 5:** Periodontal outcome reporting across preparation techniques.

Study	Technique(s)	Follow‐up	PPD	BOP/inflammation/plaque	Recession/margin stability/CAL	Risk of bias
Mohammad et al. (2023)	BOPT vs subgingival horizontal preparation	Up to 2 years	BOPT improved over time; SHPT showed increased probing depth > 3 mm.	SHPT plaque/inflammation rose markedly during follow‐up; BOPT became more favorable after 6 months.	BOPT showed better marginal stability over time. CAL was not reported in the extracted dataset.	Low
Serra‐Pastor et al. (2023)	BOPT	6 years	Stable probing depths	Low plaque levels	Gingival margin stable in 100% of cases; no recessions reported.	Moderate
Palombo et al. (2023)	BOPT vs chamfer vs unprepared teeth	4 and 12 weeks	Not reported as clinical PPD	Histologic tissue healing similar between BOPT and chamfer	Soft tissue dimensions evaluated histomorphometrically; CAL not applicable.	Low
Abad‐Coronel et al. (2022)	Systematic review of BOPT	Up to 5 years	PD > 3 mm increased by 2.3%	Gingival inflammation present in 22.8%	Recession in 1.7%; mechanical/biological failures 4.4%. CAL data were not pooled.	Review‐level

Overall, randomized studies showed a lower risk of bias, whereas non‐randomized designs —particularly case reports, expert opinions, and narrative reviews—were associated with moderate to a serious risk of bias (Table 6). For transparency, Table 7 lists the five randomized comparative studies and six controlled comparative studies requested by the reviewer. Several of these were laboratory or animal studies and therefore did not have clinical follow‐up in months; where follow‐up was not applicable or not extractable from the manuscript dataset, this is explicitly stated.

**Table 6:** Risk of bias was assessed according to study design using the RoB 2 tool for randomized controlled trials and the ROBINS‐I tool for non‐randomized studies.

Study design	Number of studies	Risk of bias tool applied	Overall risk of bias
Randomized controlled trials (RCTs)	6	RoB 2	Low
Case reports and expert opinion	8	ROBINS‐I	Serious
Narrative literature reviews	5	ROBINS‐I	Serious
Cohort studies (prospective/observational)	3	ROBINS‐I	Moderate
Systematic review	1	ROBINS‐I	Low

**Table 7:** Randomized and controlled comparative clinical studies included in the review.

Study	Design	Sample	Follow‐up	Primary outcome(s)	Key findings relevant to conservation/periodontal health
Kasem et al. (2020)	Randomized in vitro	40 maxillary premolars	Not applicable (laboratory aging only)	Fracture resistance; effect of finish line with CAD/CAM ceramics	Finish‐line type did not significantly impair strength after aging; supports feasibility of less invasive margins for selected ceramic systems.
Mancuso et al. (2022)	Randomized in vitro	50 standardized replicas	Not applicable	Enamel/dentin removal; residual adhesive surface	Chamfer was more conservative than rounded shoulder below the dental equator; margin location altered dentin exposure.
Al‐Fouzan (2016)	Randomized ex vivo	80 extracted teeth	Not applicable	Percentage of removed tooth structure	Full‐coverage preparations were more invasive; mandibular premolars lost the highest proportion of structure.
Mohammad et al. (2023)	Randomized split‐mouth clinical trial	100 patients; 2 crowns per patient	1, 3, 6 months; 1 and 2 years	Periodontal health comparing SHPT vs BOPT	SHPT showed higher plaque/inflammation over time and increased probing depth > 3 mm; BOPT improved after 6 months and remained favorable at 1‐2 years.
Palombo et al. (2023)	Randomized in vivo animal study	32 prepared teeth + 16 unprepared controls	4 and 12 weeks	Histologic and histomorphometric healing	BOPT and chamfer produced similar qualitative and quantitative healing patterns in the supracrestal tissue complex.
Edelhoff and Sorensen (2002)	Controlled in vitro	Anterior typodont teeth, 10 per group	Not applicable	Coronal tooth structure removed	Full crowns removed substantially more tissue than conservative indirect restorations.
Davis et al. (2012)	Controlled ex vivo	16 extracted incisors	Not applicable	Residual dentin thickness after crown preparation	Localized over‐reduction was common, especially at labial‐proximal line angles; this has implications for pulp safety.
Edelhoff and Sorensen (2002)	Controlled in vitro	Posterior typodont teeth, 10 per group	Not applicable	Amount of tooth structure removed	Tooth morphology materially influenced structure loss; full‐crown designs were the most invasive.
Weber et al. (2022)	Controlled in vitro	80 standardized artificial teeth	Not applicable	Influence of morphology and measurement method	Detected reduction varied by tooth morphology and by quantification method, which limits cross‐study pooling.
Gavelis et al. (2004)	Controlled in vitro	8 abutments; 5 crowns per abutment	Not applicable	Marginal seal and seating	Feather‐edge and chamfer with parallel bevel showed favorable sealing, whereas 90‐degree shoulder showed best seating.
Amesti‐Garaizabal et al. (2020)	Controlled in vivo human study	31 anterior teeth	Short‐term provisional phase; exact duration not extracted	Gingival tissue volume change with different emergence angles	A 60‐degree emergence angle increased gingival thickness and apical displacement; this informs soft‐tissue modulation rather than direct hard‐tissue conservation.

## 4 Discussion

Regarding the limitations of the included studies, it is essential to distinguish between direct quantitative evidence for hard‐tissue removal and contextual evidence concerning periodontal behavior, restorative workflows, or biologic plausibility. Most studies included in this review did not directly measure the primary outcome, and several were laboratory, descriptive, or narrative reports. This methodological heterogeneity limits external validity, prevents meta‐analysis, and requires cautious interpretation of any ranking of preparation techniques.

### 4.1 Preservation of Tooth Structure

The primary objective of this review is to investigate which dental preparation technique preserves more tooth structure in fixed unitary prosthetic restorations over teeth. The revised synthesis indicates that this question cannot be answered with a single universal hierarchy independent of margin position. Several authors agree that BOPT is the more conservative technique (Łabno and Drobnik 2020; Loi and Di Felice 2013; Kaur et al. 2022; Panadero and Pastor 2023; Kasem et al. 2020). Considering the vertical geometry of the finishing line, no margin needs to be created, thus healthy dental tissue is preserved by omitting this margin. When preparation designs are compared at an equivalent subgingival level, BOPT appears more conservative than BOPTm because BOPTm intentionally extends to the alveolar crest and removes more tissue. However, when a horizontal chamfer can be maintained in a supra‐ or juxtagingival position, that horizontal approach may remove less enamel and dentin overall than a subgingival vertical preparation, in addition showing greater exposure of the enamel bonding surface (Mancuso et al. 2022). Therefore, the interpretation is scenario‐specific rather than contradictory.

The supragingival margin in a horizontal preparation is ideal from a hygiene standpoint, while the yuxtagingival margin aims to combine esthetics and hygiene and is by far the most used in horizontal preparations. The main advantages of placing the margin outside the sulcus are: more conservative preparation, easier impression taking, easier provisional fabrication, better maintenance of gingival health, and easier removal of cement remnants (Panadero and Callís 2019). When horizontal (chamfer) and vertical (BOPT) preparation techniques are compared at the same finishing line level, available evidence consistently identifies BOPT as the most conservative option. Regarding the BOPTm technique, it can be considered the most aggressive in terms of dental structure removal, as it involves a knife‐edge preparation to the alveolar crest, completely eliminating the enamel and leaving only exposed dentin (Rodríguez et al. 2019).

### 4.2 Importance of Residual Dentin and Conservation of Dental Structure

In vitro studies have demonstrated that a supportive structure with a high elastic modulus increases the resistance of fully ceramic crowns; therefore, the residual dentin thickness after preparation may influence the restoration's longevity (Edelhoff and Sorensen 2002). Previous studies suggest that 2 mm or more are critical to prevent pulpal damage (Davis et al. 2012), as in the case of dental pulp preparation with vital dental pulp, there is a risk of irritation, inflammation, necrosis, and future endodontic treatment (Łabno and Drobnik 2020; Loi and Di Felice 2013; Kaur et al. 2022; Rodríguez et al. 2019; Higgins et al. 2023; Page et al. 2021; Sterne et al. 2019, 2016; Schünemann et al. 2023; Panadero and Pastor 2023; Kasem et al. 2020; Mancuso et al. 2022; Panadero and Callís 2019; Edelhoff and Sorensen 2002; Davis et al. 2012; Yu et al. 2019).

Although there is no consensus on the minimum thickness of dentin necessary to protect pulp vitality, it has been reported that between 2.7% and 19% of initially vital teeth develop periapical pathology after preparation for full crowns following an observation period of 1 to 25 years.16 These data highlight a clinically relevant limitation, as achieving precise dentin preservation during crown preparation remains challenging, even under controlled conditions. The present study concludes that over‐reduction (less than 2 mm of residual dentin) is more likely to occur along the labial‐proximal line angles of the teeth, when talking about anterior teeth, possibly due to the curvature of the tooth and the proximity of the pulp cusps in this region (Davis et al. 2012). From a clinical perspective, this finding emphasizes the need for enhanced operator control to minimize iatrogenic pulpal risk. It is recommended to use microscopic equipment to ensure an accuracy of 0.1 mm as well as the ergonomic comfort of the operators (Yu et al. 2019).

### 4.3 Tooth Morphology

Several studies agree that the relative amount of dental structure removed is significantly influenced by dental morphology (Al‐Fouzan 2016; Edelhoff and Sorensen 2002; Weber et al. 2022). Sorensen (Al‐Fouzan 2016) performed a horizontal shoulder termination with a rounded shoulder 0.5 mm incisal from the amelocemental junction. The first mandibular premolar showed the highest percentage of dental structure removal (40.01%), followed by the mandibular central incisor (36.49%), maxillary central incisor (30.40%), and maxillary first molar (28.33%). Another in vitro study observed that in the maxillary and mandibular tooth groups, for the same type of tooth, there is more structure removal in the mandible than in the maxilla when moving to the posterior sector (Edelhoff and Sorensen 2002).

These findings clearly indicate that dental preparation strategies cannot be standardized and must be adapted to individual tooth morphology to achieve a conservative outcome. The BOPT technique aims to eliminate the emergence profile of the tooth; therefore, the greater the discrepancy between neck width and tooth size, the more dental structure will be removed.

### 4.4 Periodontal Health

When performing subgingival preparations, inflammation and bleeding of the periodontium are frequent concerns. The strongest comparative clinical evidence in the present review comes from the split‐mouth randomized trial by Mohammad et al. (2023), in which the subgingival horizontal preparation technique showed a progressive increase in plaque, inflammation, and probing depth during follow‐up, whereas BOPT improved after 6 months and remained favorable through 2 years. At the same time, the periodontal dataset was not uniform: CAL, BOP, and recession were not consistently available, and only a small number of studies had low risk of bias. For this reason, the revised manuscript now tabulates the available periodontal indices and avoids implying that all periodontal outcomes were measured consistently across techniques.

In BOPT, there is no discrepancy between the crown and the finishing line, reducing plaque since there is no finishing line. This fact also helps reduce plaque retention in this area (Mohammad et al. 2023). We know that when we perform vertical preparations, we achieve better crown margin adaptation due to the vertical geometry, although there is greater difficulty in their seating. Conversely, when we have horizontal finishing lines, we achieve better seating but poorer adaptation (Gavelis et al. 2004). This trade‐off has direct clinical implications for marginal integrity, plaque accumulation, and long‐term periodontal stability.

This difficulty in achieving optimal adaptation may also be one of the reasons explaining the higher presence of recessions when horizontal finishing lines are performed, especially when they are located at a subgingival level (Montañes 2023). We should not automatically associate horizontal preparations with recessions. There are other crucial factors influencing the occurrence of recessions, such as periodontal health, thickness of soft tissue, tooth position, presence of plaque, amount of attached and keratinized gingiva, among others (Montañes 2023).

A thin periodontal phenotype is more prone to recession; a subgingival vertical preparation will improve the stability of soft tissues in this case (Painz et al. 2023). Findings suggest that the BOPT protocol has a potential role in promoting a phenotype increase in the submarginal portion of the supra‐crestal soft tissues surrounding the provisional restoration, as a consequence of providing more horizontal space for the supra‐crestal soft tissue complex (Palombo et al. 2023). Another advantage of the BOPT technique from a periodontal perspective is that it allows us to modify gingival contouring with the modification of the emergence of the provisional prosthesis, making it a periprosthetic technique (Mancuso et al. 2022; Amesti‐Garaizabal et al. 2020).

With greater emergence, the gingiva tends to thicken and migrate apically; with minor emergence, the gingiva tends to thin out with a coronal displacement (Amesti‐Garaizabal et al. 2020). The present study conducted by Augustin Panadero is the study that presents the maximum follow‐up duration of 6 years for BOPT cases. It concludes that full‐coverage fixed prostheses in teeth treated with BOPT have a 100% success rate, showing periodontal health, stability of the gingival margin without recessions at 6 years (Serra‐Pastor et al. 2023). Despite these highly favorable outcomes, the absence of long‐term evidence beyond 6 years remains a major limitation of the current literature.

It is crucial to note that the favorable periodontal findings for BOPT apply mainly to short‐ and medium‐term follow‐up and predominantly to subgingival clinical indications. Long‐term evidence remains limited; within the present dataset, the longest follow‐up was the 6‐year prospective cohort by Serra‐Serra‐Pastor et al. (2023) Furthermore, no included comparative trial directly reported robust pulpal outcomes such as persistent hypersensitivity, pulp necrosis, or endodontic treatment need according to preparation design. Pulp‐related considerations therefore remain indirect and are inferred mainly from residual dentin thickness studies rather than from comparative clinical endpoints.

The significance of this technique and the clinical case with histological study conducted by Rodriguez and Vela (Rodríguez et al. 2019) is that it demonstrates that despite significant sulcus invasion during the preparation and gingitage phases of the BOPT technique, regeneration will be achieved if the restoration does not invade the sulcus more than 0.5 mm.

The ideal situation would be to position the margins supragingivally to preserve dental structure and avoid damaging periodontal tissues. However, there are situations such as anterior restorations with subgingival margins, caries, crown fractures, abfraction, abrasion, chemical erosion, or color disorders that require a subgingival margin. In these cases, BOPT provides a minimally invasive preparation accompanied by periodontal health and esthetics due to the peri‐prosthetic nature of the technique.

Its disadvantages include technical sensitivity, the need for careful provisionalization and laboratory communication, and the limited amount of long‐term comparative evidence. Another important limitation is that restorative materials, CAD/CAM workflows, and scanning or impression techniques were not reported with sufficient consistency to permit stratified analysis. Because these factors may interact with margin design to affect marginal adaptation and measurable tooth reduction, future studies should control them explicitly. Accordingly, adequate training, experience, and careful case selection remain essential prerequisites for predictable outcomes. There is no universally recommended method for dental preparation for prosthetic crowns, as the choice depends on various clinical factors (Łabno and Drobnik 2020).

**Figure 4:**
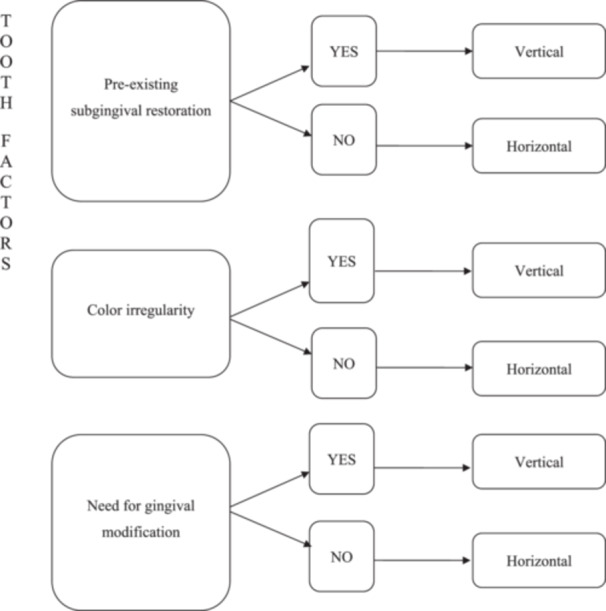
Indications for selecting margin design based on tooth analysis. Table adapted from the Literature Review by Gianluca Gavelis et al. (2004), vertical vs horizontal preparations.

**Figure 5:**
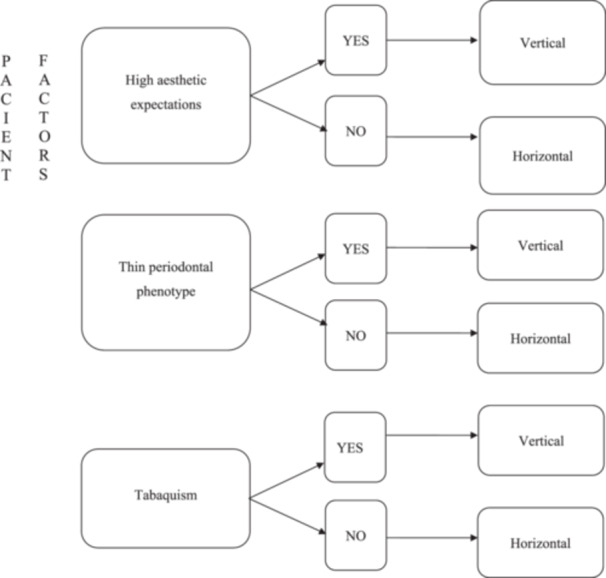
Indications for selecting margin design based on patient analysis. Table adapted from the Literature Review by Gianluca Painz et al. (22), vertical vs horizontal preparations.

Currently, clinician preference is the determining factor in margin design, but this decision is more than just a matter of preference (Painz et al. 2023). Factors influencing the choice of margin design for a full‐coverage restoration are the following: the tooth being treated (Figure 4), the patient (Figure 5) and the clinician (Painz et al. 2023). Regarding the clinician, the complexity of the vertical preparation technique BOPT in terms of provisionalization, communication with the laboratory, and cementation is recognized (Mancuso et al. 2022; Painz et al. 2023). Accordingly, adequate training, experience, and careful case selection are essential prerequisites for predictable outcomes.

**Figure 6:**
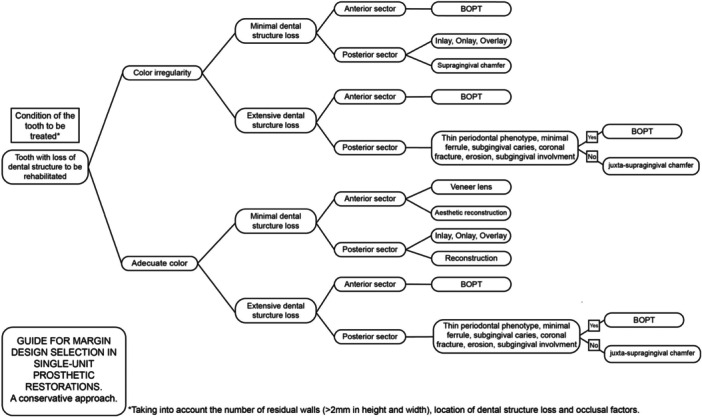
Margin design selection guide for single‐unit prosthetic restorations. Tooth with loss of dental structure to be rehabilitated.

**Figure 7:**
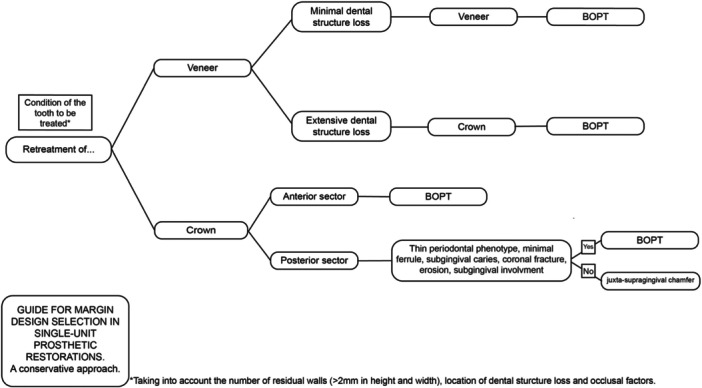
Margin design selection guide for single‐unit prosthetic restorations. Retreatmen.

Following this research, Figures 6 and 7 are proposed as a guide for choosing margin design in single‐unit prosthetic restorations with a conservative approach. This guide aims to generally present the situations in which prosthetic restoration is needed and the conservative decision‐making in each case.

## 5 Conclusions

With the limitations of the present systematic review, the conclusions were revised as follows:

- The margin location in dental preparation has significant implications for dental structure preservation, esthetics, and periodontal health. At the same subgingival margin level, BOPT appears more conservative than BOPTm because BOPTm extends more aggressively apically and is associated with greater removal of enamel and dentin. When restorative margins can be kept supra‐ or juxtagingivally, horizontal chamfer preparations remain the most conservative and clinically predictable option in terms of enamel and dentin preservation; therefore, the most conservative technique depends on the clinical scenario and margin position rather than on preparation geometry alone.
- The supragingival or juxtagingival chamfer presents as the most conservative option in enamel and dentin reduction, although its esthetic compromise may be a limiting factor, highlighting the importance of considering the balance between preservation of dental structure and esthetics in dental margin design.
- The conservation of at least 2 mm of residual dentine after tooth preparation is crucial for long‐term pulp health, as a thickness of less than 2 mm of dentine can increase the risk of pulp complications.
- The degree of dental structure removed depends on the morphology of the tooth to be prepared. When using the chamfer preparation technique, mandibular premolars tend to undergo the most removal, followed by incisors and molars. When using a BOPT‐type vertical preparation, the discrepancy between the neck width and tooth size determines the amount of dental structure removal.
- BOPT shows more favorable periodontal behavior than subgingival horizontal preparation in the limited comparative clinical evidence currently available, but standardized reporting of PPD, BOP, recession, and CAL remains incomplete across studies.
- BOPTm demonstrates that, even with sulcus invasion during preparation and gingitage, regeneration can be achieved if the restoration does not invade the sulcus by more than 0.5 mm.
- Supragingival chamfer may be preferable when periodontal conditions allow coronal margin placement, because it facilitates hygiene, impression accuracy, and removal of cement excess while minimizing hard‐tissue reduction.
- The BOPTm technique may provide increased gingival volume gain based on the same biological concepts as BOPT, but it is more aggressive from a hard‐tissue perspective and still lacks robust long‐term comparative validation.
- Direct pulpal outcomes were not adequately reported in the included comparative studies. Current pulpal inferences are therefore indirect and rely mainly on residual dentin thickness evidence, which supports minimizing over‐reduction whenever possible.

## Author Contributions

**Maria Lisa Florescu:** investigation, data curation, writing – original draft preparation. **Gelsy Arrien‐Barrenetxea:** investigation, software, formal analysis, writing – review and editing. **Cosimo Galletti:** formal analysis, validation, writing – review and editing. **Santi Costa‐Palau:** methodology, supervision, project administration, writing – review and editing. **Francisco Real‐Voltas:** conceptualization, validation, methodology, formal analysis, supervision, project administration, writing – review and editing. **Luca Fiorillo:** formal analysis, validation, writing – review and editing. **Artak Heboyan:** formal analysis, validation, writing – review and editing.

## Funding

The authors have nothing to report.

## Ethics Statement

The authors have nothing to report.

## Consent

The authors have nothing to report.

## Conflicts of Interest

The authors declare no conflicts of interest.

## Data Availability

The data supporting this study's findings are available from the corresponding author upon reasonable request.
